# Prompting Socially Shared Regulation of Learning and Creativity in Solving STEM Problems

**DOI:** 10.3389/fpsyg.2021.722535

**Published:** 2021-11-01

**Authors:** Tova Michalsky, Avigail Cohen

**Affiliations:** School of Education, Bar-Ilan University, Ramat Gan, Israel

**Keywords:** solving STEM problems, socially shared regulation of learning (SSRL), scientific creativity, middle school, collaboratively learning

## Abstract

Problem-based learning (PBL) is a widely recommended method in science, technology, engineering, and mathematics (STEM) education through which students develop their scientific knowledge by collaboratively solving real-world problems. PBL benefits from both the activation of creative thinking and from socially shared regulation of learning (SSRL)-a group-level phenomenon whereby students collectively share common perceptions of their collaborative learning process and co-construction of knowledge. The current study examines the influence of three types of support (question prompts designed to promote SSRL, creative thinking, or a combination of both) on the participation of individuals in SSRL processes and on their knowledge acquisition, using a sample of 104 seventh-graders in accelerated science classes. Individuals' participation through the different stages of SSRL (forethought, performance, and reflection) was assessed using video recordings, and their scientific knowledge was measured through pre-and post-intervention knowledge tests. While all groups improved their scientific knowledge, individuals receiving only SSRL support improved their participation in most stages of SSRL compared with those receiving creativity or combined support, and a control group which received no support. The findings strengthen the case for SSRL-directed question prompts as a means to enhance student engagement in problem-solving tasks.

## Introduction

To cope with rapid developments in the information and technology age, individuals need to adapt to innovation. This, in turn, requires “21st-century skills,” including creativity, critical thinking, research, questioning, problem-solving, and collaboration skills (Binkley et al., 2012; Nilsson and Gro, 2015; Häkkinen et al., 2017; OECD, 2018). Traditional teaching is not necessarily equipped to develop 21st-century skills (Nilsson and Gro, 2015), particularly in the teaching of science, technology, engineering, and mathematics (STEM) subjects (National Research Council, 2012a, 2015). This matters, because STEM education is key to helping students face the challenges of the 21st century and prepare them to become productive workers (Wan Nor Fadzilah et al., 2016).

An integrative framework for the teaching of STEM subjects emphasizes the value of problem-based learning (PBL), a pedagogical approach through which students develop their scientific knowledge by collaboratively solving ill-structured problems—i.e., open-ended problems allowing for multiple solutions and problem-solving paths (Kitchner, 1983; OECD, 2013; Hathcock et al., 2015; Häkkinen et al., 2017). The approach aims to develop skills, promote critical thinking, and teach scientific concepts through students' application of knowledge to practical, real-world problems. School standards which promote PBL include the U.S. Framework for K−12 Science Education (National Research Council, 2012a) and the Next Generation Science Standards (National Science Standards Education, 2013), among others.

Solving STEM problems also involves the application of creative thinking skills such as idea generation and development. Solving problems collaboratively can have an advantage over solving them individually, in that it can increase the potential for creative thinking processes to unfold (OECD, 2013). However, many things can go awry during students' collaborative work. Working collaboratively on a problem-solving task can introduce cognitive, emotional, and behavioral challenges which jeopardize the desired results (Hadwin et al., 2011; National Research Council, 2012b; Järvenoja et al., 2013).

Socially shared regulation of learning (SSRL) is a group-level phenomenon in which groups regulate their learning as a collective, for example by constructing shared task perceptions or shared goals (Järvelä et al., 2008; Hadwin et al., 2011). SSRL can be fostered in school settings, and has been shown to be effective when supported through the use of question prompts (Järvelä and Hadwin, 2013; Järvelä et al., 2015). Such support mechanisms are intended to help students overcome the cognitive, emotional, and behavioral challenges that accompany learning in groups. However, determining what sorts of support are most effective for particular objectives is an ongoing issue in educational research (Panadero and Järvelä, 2015).

In what follows, we first outline the theoretical background around our major concepts: problem-based learning, creativity, SSRL, and support. We then introduce the current study examining three types of support, using question prompts: SSRL support, Creativity support, and Combined (SSRL and Creativity) support. After analyzing the data, we conclude by discussing the findings, their implications, the limitations of the study, and suggestions for future research.

## Theoretical Background

Effective STEM education should lead students to engage with elemental questions about the world, and with approaches used by scientists to investigate and answer these questions. Learning activities in which students conduct scientific investigations and try to solve real-life problems allow young people to develop their understanding of core ideas in science and engineering, encourage them to participate in public scientific discussions, and teach them to be critical when they encounter scientific information in everyday life (National Research Council, 2012a, 2015). These goals all require scientific knowledge and scientific creativity, in the sense of being able to generate, develop and assess potential problem-solving pathways and solutions (Hu and Adey, 2002; Vincent-Lancrin et al., 2019; Bi et al., 2020). Problem-based learning in STEM education provides an outstanding opportunity to enhance students' scientific knowledge and creative processes, allowing for promotion of scientific creativity (Hathcock et al., 2015; Lyre, 2018; Bi et al., 2020).

While learning in a PBL environment, students must take responsibility for their learning process by setting goals, monitoring, and reflecting from the beginning of the task until the end. Since this process doesn't come naturally or easily for many students, support for self-regulation of learning (SRL) can help (English and Kitsantas, 2013).

### Collaborative Problem-Solving and Creative Thinking

Ayas and Sak (2014) defined scientific creativity as the ability to generate novel ideas or products that are relevant to the scientific context and have scientific usefulness or importance. Similarly, Hu and Adey (2002) defined scientific creativity as the ability to produce original products with social or personal value, designed for a certain purpose using given information. Under both definitions, solving problems in science requires students to explore their repertoire, to imagine a variety of routes to a solution, and frequently to create new combinations of knowledge or novel techniques (Hu and Adey, 2002; Hu et al., 2013).

To enhance the emergence of creativity during problem-solving exercises in class, it is important that the problem at hand be ill-structured in nature (OECD, 2013; Hathcock et al., 2015; Häkkinen et al., 2017). Having vague goals allows for multiple solutions and paths to achieve them (Kitchner, 1983), allowing students' creativity to unfold (Sullivan and Barbosa, 2017).

Solving STEM problems collaboratively also improves opportunities for creativity to emerge (Darling-Hammond, 2011; DiDonato, 2013; OECD, 2013; Häkkinen et al., 2017). By working together, individuals can combine their knowledge, effort, and understanding, allowing for meaningful creative processes (Sarmiento and Stahl, 2008; Ferreira and Dos Santos, 2009; Poutanen, 2016; Sullivan and Barbosa, 2017; Kupers et al., 2019). Indeed, many studies from the mid-1950s to today lend credence to the notion that working in a group increases the potential for creativity (Taylor et al., 1958; Larey and Paulus, 1999; OECD, 2013). However, groups can also become dysfunctional, making them less productive and less creative (Lencioni, 2002; Sawyer, 2012; Kirschner et al., 2018).

Such dysfunction can arise as the result of cognitive, metacognitive, motivational, and socio-emotional challenges (Van Den Bossche et al., 2006). Cognitive and metacognitive challenges can emerge through team members' failure to understand other members' thinking, or difficulties in negotiating multiple perspectives (Kirschner et al., 2008; Häkkinen, 2013). Motivational and socio-emotional challenges can arise due to divergence in group members' goals, priorities, and expectations (Järvelä et al., 2008). These challenges can reflect the involvement of students with different levels of knowledge, motivation, skills, and engagement in the group activity. Isohätälä et al. (2017) note that not all team members will make the same contribution to the team's work. In their study, they identified three levels of participation: active conversing (where the student contributed to the joint discussion), attunement (where the student did not substantially contribute to the joint discussion but showed signs of joint attention), and non-responsiveness (where the student did not contribute and showed few or no signs of attention). A high level of student participation increases the likelihood that SSRL processes will occur. Therefore, categorization of participation levels may come in useful when trying to understand the effect of an intervention on SSSL.

### Regulation of Learning

Regulation of learning is an intentional process in which individuals take control of their own thinking (cognition), actions (behavior), and beliefs (motivation, emotions) to successfully complete a learning task (Zimmerman and Schunk, 2011). The challenges inherent in collaborative work mean that group-level regulation of learning, or SSRL, becomes a necessary component of successful collaborations. SSRL involves interdependent or collectively shared regulatory processes, beliefs, and knowledge (e.g., strategies, monitoring, evaluation, goal-setting, motivation, and metacognitive decision making), orchestrated in the service of a co-constructed shared knowledge or other shared outcome (Hadwin et al., 2011). Earlier studies have pointed out the close relationship between learners' active participation and manifestation of the regulation process during interactions (Rogat and Linnenbrink-Garcia, 2011; Grau and Whitebread, 2012; Sinha et al., 2015). However, regulation of this sort usually does not occur spontaneously, and the complexity of generating, developing, and maintaining it while collaboratively completing the task may lead to negative learning experiences, in which group members may fail both to effectively carry out the task and to interact productively in their group (Järvelä et al., 2016). More precisely, learners may fail to plan adequately, to use adaptive learning strategies, to collaborate, and to competently complete problem-solving tasks (Zimmerman and Schunk, 2011; Järvelä and Hadwin, 2013). To mitigate this problem, teachers can apply various regulatory tools to support students as they develop and strengthen their competence in group processes (Järvelä and Hadwin, 2013; Hathcock et al., 2015; Järvelä et al., 2015; Panadero and Järvelä, 2015; Van Merriënboer and Kirschner, 2017).

### Effectiveness of Using Question Prompts for SSRL, PBL, and Creativity

Support is defined here as an educational technique aimed at enabling learners to accomplish tasks which otherwise might have been too challenging by, in part, making the task cognitively easier (Rosenshine and Meister, 1992; Hathcock et al., 2015). Targeted support can help promote co-construction of shared knowledge and enhance the quality of the solution ultimately reached (Barron, 2009; Belland et al., 2013; Molenaar et al., 2014).

One method of support which has been widely researched and found effective is the use of question prompts-for example, “What is the goal in this task?” or “What information do I need to find a solution to this problem?” (Xie and Bradshaw, 2008; Zheng et al., 2013; Hathcock et al., 2015). Question prompts designed by teachers can help students regulate and improve their learning when engaged in tasks by guiding them to justify their choices, explain their reasoning, evaluate their decisions, and better understand the kinds of questions which should be addressed (Xie and Bradshaw, 2008). The use of question prompts has been found to be effective in promoting self-(Michalsky, 2013) and socially shared regulation of learning (Järvelä et al., 2016), problem-solving (Ge and Land, 2003; Hmelo-Silver et al., 2007), and creativity (Zheng et al., 2013; Vincent-Lancrin et al., 2019).

As noted above, STEM studies encompass an extensive range of activities and processes, such as problem-solving and idea generation, which mutually promote and benefit from creativity (Al-Abdali and Al-Balushi, 2016; Schlatter et al., 2020). Several studies show a positive effect of training on scientific creativity. For example, in a study with 105 eleventh-grade students in China, Sun et al. (2020) showed that students' scientific creativity performance improved after training. Notably, they also found that students with high and low levels of creative potential benefited equally from the training. Sun et al. argued that creativity training can facilitate divergent thinking by making cognitive processes explicit to learners. Such findings join a large body of work pointing to the benefits of creativity in education more generally (Bryan-Kinns, 2012; Lucas et al., 2013; de Vries and Lubart, 2019; Vincent-Lancrin et al., 2019), and have led to growing interest over the past decade in research on ways to promote creative thinking in STEM education (Lucas et al., 2013; Barrett et al., 2015; Al-Abdali and Al-Balushi, 2016; Sullivan and Barbosa, 2017).

### The Current Study

The current study responds to growing recognition of the links between collaboration, problem-solving, SSRL, and creative thinking in STEM education, and the potential use of support via question prompts during these processes. As mentioned above, many studies point to the advantages of supporting both collaborative work (Xie and Bradshaw, 2008; Järvelä et al., 2015, 2016) and creativity (Zheng et al., 2013; Hathcock et al., 2015) as students engage in problem-solving tasks. However, there is little understanding of how support can best be directed-whether toward SSRL, toward creativity, or toward some combination of both. Moreover, to the best of our knowledge, no study has yet examined the effects of combined support for these two aspects of problem-solving, collaboration and creativity, particularly in STEM education. Examining the two types of support separately and together can shed light on what types of support are most effective in promoting problem-solving in STEM education.

The present study aimed to compare the influence of three types of support on group members' learning regulation and scientific knowledge. One type of support was specifically designed to support the regulation of learning (SSRL), the second was specifically designed to support creative thinking, and the third comprised a combination of the two, creating three experimental groups: SSRL, Creativity, and Combined. Eight classes of students were randomly assigned to one of the three experimental groups or a control group (two classes each).

In accordance with previous studies in the field of individual self-regulation (e.g., Dignath and Büttner, 2008), our framework for all three experimental groups followed the Zimmerman (2000) cyclical model of self-regulation of learning. This model holds that interventions should aim to promote the three stages of task execution: forethought, performance, and reflection. All three experimental groups received written question prompts, along with verbal support from the students' teachers, who were trained for this purpose (see under Procedure, below). Broadly speaking, the SSRL support was based on theoretical research into collaborative regulation of learning, and in particular metacognitive awareness of cognitive, motivational, and emotional group-level regulation processes (Michalsky, 2013; Järvelä et al., 2015, 2016; Michalsky and Kramarski, 2015). The Creativity support was devised based on Torrance's (1965) three components of creativity: fluency, capturing the number of possible solution ideas generated; flexibility, capturing the number of different categories into which possible solutions fall; and originality, capturing the number of responses which are statistically infrequent. The SSRL and Creativity experimental groups each received the relevant form of support, and the Combined group received both types of support.

This study examined two outcomes: (1) the level of participation of group members in SSRL during the three stages of group problem-solving tasks, namely forethought, performance, and reflection (a qualitative measure), and (2) their scientific knowledge following completion of the tasks (a quantitative measure). The analysis was guided by the following research questions: (RQ1) How do the four study groups differ in their level of participation during the three stages of SSRL (forethought, performance, and reflection) before vs. after the intervention? (RQ2) How do the four study groups differ in their scientific knowledge before vs. after the intervention?

## Methods

### Participants

The participants of this study were 104 seventh-grade students aged 12–13 from eight middle schools in Israel (51 girls, 53 boys). The schools were similar in size, with seven classes per grade. All eight schools serve populations of middle-class socio-economic status as defined by the Israel Central Bureau of Statistics (2016), and achieve average scores on Israeli national standardized tests. Eight classes, one from each of the eight different schools, were involved in a science acceleration program, in which extra school hours (90 min once a week) were allocated for science studies. Students in these classes were selected from within their grades after receiving high marks on internal tests administered by the schools. Six of these classes were randomly assigned to the three experimental groups (SSRL, Creativity, and Combined; two classes per group), while the remaining two classes served as the control group and received no support.

The eight classes together included 135 students. For the purposes of the study, each class was divided randomly into work teams, with three to four students in each team, producing 40 teams. Data for nine of these teams were dropped (four teams of four students, and five teams of three) from the analyses because of student absences during the study period. This produced a final study sample of 104 students in 31 teams (11 teams of four students, and 20 teams of three). Altogether, there were 27, 30, 23, and 24 participants in the SSRL, Creativity, Combined, and control groups respectively^1^.

The study was approved by the Research Ethics Board at Bar-Ilan University, and by the Chief Scientist in the Israeli Ministry of Education (permit number 9,341). In addition, all students provided signed parental consent forms, and all teachers signed consent forms, before the start of the study.

### Procedure

Before the beginning of the study, the eight teachers who ran the classes attended teacher training according to their respective treatment group. The training was conducted one-on-one and led by one of the authors of this study. All eight teachers first attended a 3-h basic training session covering (a) the importance of enhancing students' scientific creativity, (b) the problem-solving tasks that would be assigned during the study, (c) difficulties that can arise when encountering such tasks, and (d) the pedagogical content relevant to the study unit (on energy; see below). The six teachers in the three experimental groups then received extra training as appropriate. The two teachers leading the SSRL support classes received training in the rationale and techniques of the SSRL guidance method they would be implementing, as well as methods to model and introduce the subject. The two teachers leading the Creativity support classes were given an introduction to creativity comprising different definitions and approaches, techniques for teaching scientific creativity, and its connection to the curriculum and to the energy unit in particular. The two teachers who led the Combined group were trained in all topics. The extra training lasted about 3 h each for the teachers in the SSRL and Creativity groups, and 5 h for the Combined group.

In these extra training sessions, the prompts to be used during the study were presented along with the rationale behind implementing them throughout the task. Teachers were told to encourage the teams to use these prompts, and to model the use of the prompts when they introduced the tasks to their classes or when helping students. The teachers were also encouraged to initiate verbal instructions to their students—e.g., “Discuss terms in energy that appear in your solution with your teammates,” or “Try to think about different ideas that your fellow teammates are raising.”

Students in all eight classes spent three lessons learning a unit on energy as part of their regular syllabus. For the study, as described above, each class was divided randomly into work teams. Then, the teams were given a series of five collaborative scientific problem-solving tasks broadly based on the energy unit the students had learned prior to the study. Each task was designed to be completed in one 90-min session, with one task assigned each week^2^. The tasks were handed to the teams as worksheets. All five tasks were similar in structure, and contained a problem scenario, a challenge, and a set of instructions to guide students' work on the task. A sample scenario with its associated challenge and instructions can be found in the Appendix.

The problem scenarios for the tasks were ill-structured, meaning that they allowed for a range of problem-solving pathways and solutions. All the scenarios were rooted in the classes' science and technology curriculum, and were designed such that the students could make use of knowledge they had learned previously (in particular in the energy unit they had learned prior to the study), but would have to build on that knowledge independently to come up with solutions. The instructions that accompanied each scenario were designed to guide students through the different stages of the task (e.g., “Come up with as many solutions as possible. Describe in detail two of your ideas,” and “Describe three terms, principles, or phenomena with which you may be familiar from science class that came up in the solutions which you suggested”). The support question prompts used for the experimental groups were presented separately from these instructions (see under Intervention, below). The sample scenario in the Appendix shows the question prompts in speech bubbles.

The first and fifth tasks were used for pre-and post-intervention assessments. These two tasks each consisted of two parts. In the first part, the teams read the scenarios, worked through the worksheet, and came up with solutions. In the second part, each team built a model of its chosen solution from a set of materials recycled from common household goods (e.g., cardboard boxes and paper towel rolls). Both parts took place during the 90-min class, proceeding at the team's own pace. Teams were video-recorded using GoPro cameras with wide lenses and equipped with external microphones for audio enhancement.

The second, third, and fourth tasks were used for the intervention and included support for the experimental groups. These tasks included only the first part of tasks one and five described above (i.e., the teams read the scenarios, worked through the worksheet, and came up with solutions, but did not build models). In these tasks, the three experimental groups received support in the form of prompts printed on their worksheets (see under Intervention, below). The task order was shuffled between teams to prevent systematic order effects.

As described above (see under Participants), a total of 40 teams were initially video-recorded. Of these, nine teams were dropped from the analyses due to student absentees in the teams during one or more of the subsequent four tasks. Videos from the remaining 31 teams (20 h 31 min, Mduration = 40 min, Std = 3 min) were used for the micro-level analysis. The period coded and analyzed in each video was shorter than the actual duration of group work filmed, due to students moving around the class while building their models, students blocking the cameras and microphones, etc.

All students took a scientific knowledge test a week before the start of the study, and a similar test a week after the final task (see under Data analysis, below). The study procedure is summarized in Table 1.

**Table 1:** Summary of the study procedure.

**Week number**	**Research stage**	**Activity**			
1-2	Before experiment/study preparation	• Classes randomly assigned to study groups			
		• Signed parental consent forms collected			
		• Teacher training by study group			
		• Classes divided into work teams of 3 to 4 students in each team			
		• Pre-intervention scientific knowledge test			
3	Pre-intervention: first problem-solving task	Sessions recorded for pre-intervention SSRL analysis (no support)			
4	Intervention: second, third and fourth problem-solving tasks	SSRL support	Creativity support	Combined support	No support (control)
5					
6					
7	Post-intervention: fifth problem-solving task	Sessions recorded for post-intervention SSRL analysis (no support)			
8	After experiment/study closure	Post-intervention scientific knowledge test			

### Intervention

During the three intervention tasks (the second, third, and fourth tasks), the three experimental groups received support in the form of question prompts printed on their worksheets. These prompts were printed separately from the regular worksheet questions described above, and were designed to focus students' attention on the process of working collaboratively or creatively. For example, in the forethought stage the SSRL group received the question prompt “How do you plan to work cooperatively in your team?,” while the Creativity group received this one: “How can you increase the number of ideas to solve the problem?” The Combined group received both sets of prompts. Prompts were included for all three stages of the process (forethought, performance, and reflection) (see Appendix). The study groups were also supported directly by their teachers.

### Data Analysis

The effect of the intervention was assessed through two measures, qualitative and quantitative. The former addressed RQ1, and the latter addressed RQ2.

#### Qualitative Analysis (RQ1)

Video analysis was conducted to assess group-and individual-level participation in SSRL, using the Observer XT video analysis software platform (Noldus). Following Isohätälä et al. (2017), we used a systematic threshold of 20 s. This time threshold was used because it permitted momentary variation in participation and enhanced the uniformity of the analysis. Each segment was coded in two rounds, where the first concentrated on group interaction and the second on level of participation.

##### Round One: Group Interaction

The purpose of round one was to examine students' interactions within their teams. First, students' interactions were identified as either task-directed or not. Task-directed behavior could be either verbal or non-verbal (e.g., gestures and actions), as long as these were pertinent to the task. Segments in which the team was engaged in activities not pertinent to the task—e.g., playing with the microphones or camera, discussing other coursework, talking about their personal lives, etc.—were coded as “off-task.” Segments were only coded as off-task if the team members engaged in non-pertinent activities for the entire 20-s segment. Segments were identified as task-directed as long as collaborative task-directed behavior was observed at least once in the 20-s segment.

Segments in which the team was seen to be working collaboratively on the task were coded based on Zimmerman's (2000) SRL theory and the cyclical model of self-regulation (Cleary and Zimmerman, 2012). Specifically, segments were coded as reflecting either forethought, performance, or reflection. These categories were mutually exclusive and could not overlap. Examples of coding based on the SRL cyclical model can be seen in Table 2.

**Table 2:** Example of data coding.

**Group process**	**Category**	**Examples**
Forethought	Processes activated in preparation for the learning itself. These include task analysis (goal setting and strategic planning) and self-motivational beliefs (expectations, interests, etc.).	Motivation (e.g., “We should come up with the best idea in the class”); dividing the work (e.g., “Each team member should read one question and write the group's answer”); discussing challenges (e.g., “We need to leave enough time to build the model”).
Performance	Processes that occur during learning efforts. These include self-control (learning to focus on the task and use strategies to achieve goals) and self-observation (monitoring specific aspects of performance).	Praising a solution (e.g., “Wow, this is a great idea, let's develop it”); writing together (e.g., “We should write a complete answer that includes the relevant component of the question”); asking for help from the teacher (e.g., “Let's ask if the photoelectric effect means that the sun hit the panel or every light”).
Reflection	Evaluating the team's behavior against the goals that were set at the beginning of the task, and making changes if necessary.	Discussing feelings aroused by the task (e.g., “Not everyone shared their ideas, maybe we should raise more ideas before continuing to build the model”); praising the group for a good session (e.g., “Our idea is very smart”).

The reliability of the coding was checked by double-coding the collaborative behavior of six randomly selected teams (out of the 31). Cohen's kappa values pointed to high inter-rater reliability, with *k* = 0.94 (Std = 0.05) for segments coded as off-task, *k* = 0.86 (Std = 0.40) for forethought, *k* = 0.96 (Std = 0.15) for performance, and *k* = 0.89 (Std = 0.19) for reflection.

##### Round Two: Level of Participation

The purpose of round two was to examine the level of participation of team members within the broader categories of forethought, performance, and reflection identified above (by definition, there was no participation in the off-task category). This was the main coding process used for the later analysis. The coding was based on previous work by Isohätälä et al. (2017), who scored level of participation using three mutually exclusive categories: active conversing, attunement, and non-responsiveness. In the present study, we coded each student's behavior throughout each 20-s segment, awarding 0–3 points depending on the student's behavior. During active conversing (three points), the student verbally contributed to the group's discussion, either by initiating turns or responding to turns. Attunement (two points) was defined as showing signs of attention through back-channeling (e.g., “uh-huh”) or non-verbal reactions (e.g., laughing, leaning in, eye contact, attentive gaze on a common object of attention). Students were coded as non-responsive (one point) when they did not contribute and showed little or no signs of attunement while other members of the team worked collaboratively. In segments coded as off-task, all team members received 0 points. Each individual's scores for the full set of 20-second segments were then averaged separately for each stage (forethought, performance, and reflection) of each part of the task (the first and the second) in the pre-intervention and post-intervention assessments, thus creating twelve scores for each participant.

The reliability of the coding was checked by double-coding the participation scores from the six randomly selected teams used for the reliability checks in round one. Cohen's kappa values pointed to high inter-rater reliability, with *k* = 0.91 for segments coded as one point (Std = 0.22), *k* = 0.90 (Std = 0.07) for two points, and *k* = 0.97 (Std = 0.07) for three points.

#### Quantitative Analysis (RQ2)

All participants completed a pre-and post-intervention scientific knowledge test taken from the science and technology section of Israel's national standardized tests, which are approved by the head of STEM in Israel's Ministry of Education. This multiple-choice test comprised ten questions regarding energy and energy transfer (different sets of questions were used in the pre and post-tests). Analysis of variance (ANOVA) was used to examine the differences between the four study groups in scientific knowledge before and after the intervention (see under Results below).

Before addressing the research questions, we first examined whether the dependent variables were normally distributed by conducting Shapiro-Wilk tests for each study group. The dependent variables deviated significantly from normal distribution (*p* < 0.05). Therefore, we conducted both non-parametric and parametric analyses. For the former, we used the Wilcoxon test to compare the two time points for each study group, and the Kruskal-Wallis test to compare the four study groups at each time point. In what follows, we report the results for these non-parametric tests only where they differ from the results for the parametric analyses. Two-way mixed ANOVA (2 × 4) analyses with study group as a between-subjects factor and time point as a within-subject factor were conducted to examine both research questions.

## Results

The parametric analysis was conducted through a two-way mixed two (time: pre and post) × 4 (group: SSRL, Creativity, Combined, Control) ANOVA with group as a between-subjects factor and time as a within-subject factor. In addition, we conducted one-way ANOVAs to test for differences between the four study groups in some of the dependent variables before the intervention, and one-way ANCOVAs to test for differences between the four study groups after the intervention, while controlling for the pre-intervention measures. The results of the one-way and two-way analyses were highly similar. Therefore, in what follows we report the results only for the two-way mixed ANOVAs. In cases where the interaction of group and time was significant, we also present the results of paired samples *t*-tests and effect sizes (Cohen's *d*).

In what follows, we first report the results of the qualitative analysis (RQ1) and then the results for the quantitative analysis (RQ2).

*RQ1: How do the four study groups differ in their level of participation during the three stages of SSRL (forethought, performance, and reflection) before vs. after the intervention?*

As will be recalled, level of participation was scored by coding students' behavior in the video recordings described above. To test for differences between the four study groups in level of participation, we conducted two-way mixed ANOVAs with time (before and after) as a within-subject factor and group (SSRL, Creativity, Combined, control) as a between-subjects factor, comparing participation scores in the pre-intervention assessment (task one) and the post-intervention assessment (task five). In each case, we conducted separate analyses for the different stages of SSRL (forethought, performance, and reflection). In addition, as will be recalled, the first and fifth tasks each had two parts, part one and part two. We therefore conducted two separate sets of these analyses, one set for the first part of the task (see Table 3) and the other for the second part of the task (see Table 4). In what follows, we report the results for the two parts of the task separately.

**Table 3:** Means, SD and *F*-values for participation in different stages of SSRL during the first part of the task by group and time.

		**Before**		**After**			***F*** **-values (** **η_***p***_** ^**2**^ **)**		
	**Groups**	***M***	***SD***	***M***	***SD***	**Cohen's *d***	**Group**	**Time**	**Group × Time**
Forethought	SSRL (*n* = 27)	0.79	1.28	2.66	0.37	1.40	7.32^***^ (0.18)	37.95^***^ (0.27)	2.76^*^ (0.08)
	Creativity (*n* = 30)	0.90	1.23	1.77	1.16	0.42			
	Combined (*n* = 23)	1.39	1.29	2.14	1.01	0.41			
	Control (*n* = 27)	0.59	1.04	1.22	1.31	0.48			
Performance	SSRL (*n* = 27)	2.36	0.37	2.67	0.18	0.96	4.24^**^ (0.11)	1.20 (0.01)	3.87^*^ (0.10)
	Creativity (*n* = 30)	2.33	0.35	2.23	0.46	0.22			
	Combined (*n* = 23)	2.38	0.33	2.48	0.30	0.31			
	Control (*n* = 27)	2.29	0.42	2.19	0.59	0.13			
Reflection	SSRL (*n* = 27)	1.74	1.33	2.41	0.84	0.39	1.53 (0.04)	4.86^*^ (0.05)	0.50 (0.02)
	Creativity (*n* = 30)	1.45	1.26	1.77	1.13	0.22			
	Combined (*n* = 23)	1.75	1.25	1.84	1.21	0.06			
	Control (*n* = 27)	1.79	1.26	2.12	1.01	0.19			

* *p < 0.05*,

** *p < 0.01*,

*** *p < 0.001*.

**Table 4:** Means, SD and *F*-values for participation in different stages of SSRL during the second part of the task by group and time.

		**Before**		**After**			***F*** **-values (** **η_***p***_** ^**2**^ **)**		
	**Groups**	***M***	***SD***	***M***	***SD***	**Cohen's *d***	**Group**	**Time**	**Group × Time**
Forethought	SSRL (*n* = 27)	2.76	0.48	2.73	0.33	0.06	11.12^***^	1.38	0.57
	Creativity (*n* = 30)	2.05	0.92	1.68	1.13	0.24	(0.26)	(0.01)	(0.02)
	Combined (*n* = 23)	2.16	1.10	2.18	1.01	0.01			
	Control (*n* = 24^a^)	2.45	0.60	2.26	0.57	0.23			
Performance	SSRL (*n* = 27)	2.41	0.56	2.66	0.24	0.49	3.50^*^	0.09	4.82^**^
	Creativity (*n* = 30)	2.31	0.49	2.13	0.62	0.28	(0.10)	(0.00)	(0.13)
	Combined (*n* = 23)	2.35	0.50	2.47	0.41	0.25			
	Control (*n* = 24^a^)	2.39	0.36	2.13	0.53	0.50			
Reflection	SSRL (*n* = 27)	1.00	1.35	2.27	1.09	0.60	3.21^*^	12.48^***^	1.77
	Creativity (*n* = 30)	0.68	1.17	1.09	1.27	0.24	(0.09)	(0.11)	(0.05)
	Combined (*n* = 23)	1.12	1.36	1.41	1.39	0.11			
	Control (*n* = 24^a^)	0.83	1.23	1.40	1.18	0.48			

* *p < 0.05*,

** *p < 0.01*,

*** *p < 0.001*;

a *Three students in the control group did not participate in the second part of the task*.

### First Part of the Task

As shown in Table 3, during the first part of the task, significant interactions of group and time were found for both the forethought and performance stages. With respect to *forethought*, paired samples *t*-tests show significant differences between the two time points in all four study groups [SSRL: *t*_(23)_ = 6.85, *p* < 0.001, *d* = 1.40; Creativity: *t*_(29)_ = 2.28, *p* = 0.030, *d* = 0.42; Combined: *t*_(22)_ = 1.96, *p* = 0.050, *d* = 0.41; and control: *t*_(26)_ = 2.50, *p* = 0.019, *d* = 0.48]. Thus, all students, regardless of their study condition, participated more during the forethought stage after completing the intervention compared with before the intervention. However, as can be seen, the effect size for this difference was significantly higher in the SSRL group compared to the other three groups.

With respect to the *performance* stage, a significant difference between the two time points was found only among students in the SSRL group, *t*_(23)_ = 4.70, *p* < 0.001, *d* = 0.96. In other words, participation during the performance stage was significantly higher after the intervention compared to before the intervention only in the SSRL group.

In the *reflection* stage, a significant main effect of time was found, indicating that participation in the reflection stage was significantly higher after the intervention (*M* = 2.02, *SD* = 1.07) compared to before the intervention (*M* = 1.67, *SD* = 1.26). No interaction was found between time and group in the reflection stage.

### Second Part of the Task

As Table 4 shows, a significant interaction of group and time was found only for the *performance* stage. Paired samples *t*-tests show a significant difference between the two time points among students in both the SSRL group and the control group, *t*_(23)_ = 2.42, *p* = 0.024, *d* = 0.49, and *t*_(23)_ = 2.44, *p* = 0.023, *d* = 0.50, respectively. However, while in the SSRL group participation in the performance stage was significantly higher after the intervention compared to before the intervention, in the control group participation in the performance stage was significantly *lower* after the intervention compared to before it.

Regarding the *forethought* stage, neither a main effect of time nor an interaction of time and group were found. By contrast, we found a significant main effect of group. Scheffe *post-hoc* analysis indicated higher participation in the forethought stage among students in both the SSRL group and the control group compared to students in the Creativity group (*p* < 0.001 and *p* = 0.022, respectively).

Regarding the *reflection* stage, no significant interaction was found in the ANOVA analysis. However, Wilcoxon tests showed a significant difference between the two time points among students in the SSRL group, Z = 2.55, *p* = 0.011, but not among students in the other three groups (Creativity: Z = 1.24, *p* = 0.216; Combined: Z = 0.39, *p* = 0.698; and control: Z = 1.90, *p* = 0.064).

*RQ2: How do the four study groups differ in their scientific knowledge before vs. after the intervention?*

To answer RQ2, we tested for differences between the four study groups in students' scores on scientific knowledge tests taken before and after the five study tasks. Specifically, we conducted two-way mixed ANOVAs with time (before and after) as a within-subject factor and group (SSRL, Creativity, Combined, control) as a between-subjects factor. The dependent variable was the students' scientific knowledge test scores.

**Figure 1:**
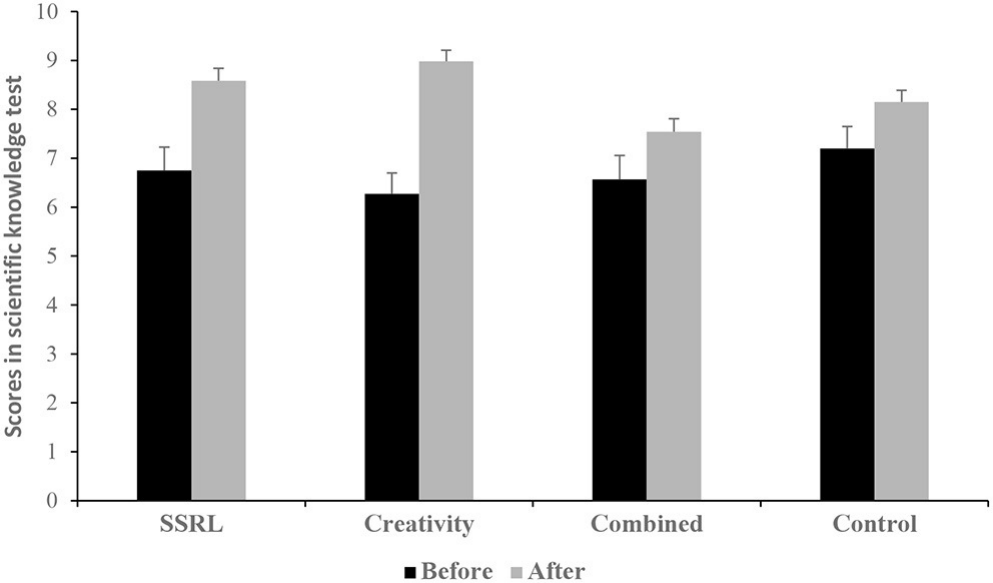
Means (and SE) of the scores in the scientific knowledge test by group and time.

The main effect of group was not significant, *F*_(3, 100)_ = 0.95, *p* = 0.420, η_*p*_^2^ = 0.03. By contrast, the main effect of time was significant, *F*_(1, 100)_ = 51.65, *p* < 0.001, η_*p*_^2^ = 0.34, indicating higher scores in the scientific knowledge test after the intervention compared to before the intervention. Finally, the two-way interaction of group and time was significant, *F*_(3, 100)_ = 3.75, *p* = 0.013, η_*p*_^2^ = 0.10 (see Figure 1).

Paired samples *t*-tests revealed significant differences between the two time points in all four study groups [SSRL: *t*_(23)_ = 3.09, *p* = 0.005, *d* = 0.63; Creativity: *t*_(29)_ = 6.34, *p* < 0.001, *d* = 1.16; Combined: *t*_(22)_ = 2.88, *p* = 0.009, *d* = 0.60; and control: *t*_(26)_ = 2.39, *p* = 0.024, *d* = 0.46]. Thus, scores in the scientific knowledge test improved from the pre-to post-intervention test among all four groups. However, comparing the effect sizes shows that the improvement was significantly greater in the Creativity group compared to the other three groups.

Finally, Pearson correlation analyses were conducted to test for a relationship between students' level of participation in the SSRL stages and their scientific knowledge scores before the intervention. A significant positive correlation was found between students' pre-intervention scientific knowledge scores and their level of participation in the reflection stage of the first part of the task, *r*_(102)_ = 0.30, *p* = 0.002. In other words, students who exhibit greater scientific knowledge scores also participate more actively in reflecting about the ideas their team developed in response to the challenge outlined in the task scenario. No significant correlations were found between the students' scientific knowledge scores and their level of participation in the forethought and performance stages in the first part of the task [*r*_(102)_ = 0.07, *p* = 0.449 and *r*_(102)_ = −0.01, *p* = 0.954, respectively]. In addition, no significant correlations were found between students' scientific knowledge scores and their level of participation in any of the stages during the second part of the task [*r*_(102)_ = −0.03, *p* = 0.786, *r*_(102)_ = 0.04, *p* = 0.698 and *r*_(102)_ = 0.18, *p* = 0.074 for the forethought, performance, and building stages, respectively].

## Discussion and Conclusion

### The Advantages of Supporting SSRL

There is extensive recognition that new methods for enhancing collaborative problem-solving are required to promote 21st-century skills. Previous studies showed the positive effects of supporting groups' regulation processes as they engage in different tasks (Järvelä and Hadwin, 2013; Järvelä et al., 2015; Järvenoja et al., 2017), and specifically in problem-solving tasks (Ge, 2001; Ge and Land, 2003; Belland et al., 2013; Liu et al., 2021). The findings of the current study likewise point to the benefits of receiving support for SSRL on the level of participation while engaging in collaborative problem-solving tasks. More specifically, we found that such a support improved the level of participation in nearly all the stages of learning regulation examined (forethought and performance in the first part of the task; forethought, performance, and reflection in the second part of the task). The other study groups displayed either lower improvement or, more often, no improvement in individuals' socially shared regulation of learning over the course of the study.

As for the differences between the groups, it could be noticed that the SSRL group improved their level of participation across the board, at all stages (from forethought in part one of the task, through performance in parts one and two, and finally reflection at the end of part two of the task). This is opposed to the control group, which showed a deterioration in their level of participation at the performing stage, the heart of the task execution. These findings are in line with previous studies showing that when support is not provided, not only does performance not improve, but negative interactions can arise within the group, threatening the collaboration needed to complete the task (Järvelä et al., 2016). Interestingly, the Combined group, which received both types of prompts, did not improve their level of participation despite receiving prompts for SSRL. Indeed, the Combined group performed substantially worse than the SSRL group despite receiving the same prompts. It may be that the combination of SSRL and Creativity prompts led to an overflow of information that did not allow participants to fully incorporate the relevant prompts into their work. That is, the high overall number of prompts for the Combined group may have been counterproductive, overwhelming participants rather than supporting them (Michalsky, 2013).

The question arises as to why support provided to the SSRL group in the current study was so effective in terms of students' participation levels relative to support for creative thinking. The prompts used in the SSRL group directly confronted the teams with different challenges and difficulties (cognitive, motivational, behavioral, etc.) that can arise during collaborative work, while also encouraging the teams to discuss the strengths and capacities of each team member. This contrasted with the Creativity group, whose support questions emphasized the creative process and not the group dynamic.

Results of previous studies have shown that SSRL is most effective when all participants of a group, rather than only some, are attuned to each other's contributions (Isohätälä et al., 2017). The current study strengthens this finding, as adding SSRL support had a positive influence on group interaction. Each team member brings his or her own learning strategies, challenges (cognitive, motivational, behavioral, etc.), and capabilities, which influence the group's dynamic and capacity to achieve its goals (Hadwin et al., 2011; Järvelä and Hadwin, 2013; Panadero and Järvelä, 2015). Moreover, shared regulation was found to be more common in groups with more active participation (Rogat and Linnenbrink-Garcia, 2011; Grau and Whitebread, 2012; Sinha et al., 2015). High levels of participation allow reciprocal exchanges, strengthening the collaborative processes which are necessary for SSRL (Iiskala et al., 2011). Ucan and Webb (2015) noted that episodes of shared regulation occurred when attentive listening happened and openness to divergent ideas was marked.

However, interestingly, while all the experimental groups displayed improvement in their scientific knowledge scores, the Creativity group improved the most. The improvement of all four groups in this measure is in line with previous studies, which showed that placing students in a problem-based learning environment—especially when they must construct their knowledge through exchanges with others—improves their academic achievement compared to traditional teaching methods (e.g., Sungur et al., 2006). For this reason, policymakers support the implementation of problem-based learning in order to promote academic achievement, both in general (OECD, 2013) and in STEM education in particular (National Research Council, 2015). Yet the fact that the Creativity group showed the highest improvement is notable. As discussed earlier, solving problems in science requires students to explore new combinations of knowledge and try a variety of routes to a solution (Hu and Adey, 2002; Hu et al., 2013)—all of which require creativity. And indeed, creativity is positively associated with both academic achievement in general (Gajda et al., 2017) and scientific knowledge (Huang et al., 2017). Thus, our findings support the notion that science education should aim to improve students' creative thinking alongside their factual scientific knowledge.

We also found a positive relationship between scientific knowledge scores in the pre-intervention test and level of participation in SSLR during the reflection stage in the first part of the task. This finding is in line with previous studies, which have also found a relationship between SSRL and knowledge co-construction (Volet et al., 2009) and between SSRL and scientific achievement (Lin et al., 2015).

The present findings suggest practical implications for STEM education programs targeting middle-school students. Empowering students' SSRL and creativity through an appropriate support framework, one that includes question prompts, has great potential to influence students' scientific knowledge growth.

In particular, the SSRL group showed improvement in different stages of the SSRL process, while the Creativity group had a significant advantage in improved scientific knowledge scores. Barron et al. (1998) and Davis (2003) point to the importance of designing support programs carefully in light of the learning objective. The different types of support tested in the current study targeted different objectives in collaborative problem-solving tasks: support directed specifically toward SSRL, support directly toward creativity and a combination of both. Thus, there is no “one size fits all” support system. Rather, educators tasked with designing a support program should ask “When to support?,” “How to support?” and “Whom or what to support?” (Azevedo and Jacobson, 2008).

The current study has a number of limitations, which also offer scope for further research. First, we focused only on how support in the form of question prompts influences learning processes and scientific knowledge. Contemporary creativity research explores socio-cultural aspects of the subject, including collaborative creativity (Sawyer and Dezutter, 2009; Poutanen, 2016). Future research could examine the effects of other kinds of support interventions on teams' creative processes, as well as on individual creative thinking.

Second, the research group was comprised of seventh-grade students in science enrichment classes. To enroll in these classes, students underwent a selection process which required relatively high grades, motivation, and an interest in STEM. However, creativity and cooperation skills are important for all students. Future research should enlarge the scope of the study with a more heterogeneous population of students, including those with average STEM grades and different age groups.

Finally, the number of students, tasks and scenarios was limited. The study was conducted over seven weeks: 5 weeks of intervention and 2 weeks for pre-and post-intervention tests. Tests or observations after a significantly longer time period (e.g., 6 months post-intervention) were beyond the scope of this study. Clearly, there is a need for closer examination of how the types of support used in this study affect group dynamics and other outcomes for both individuals and groups over time, in terms of levels of participation, regulation of learning, and creative thinking processes.

## Data Availability Statement

The raw data supporting the conclusions of this article will be made available by the authors, without undue reservation.

## Ethics Statement

This study was reviewed and approved by Bar-Ilan University's Institutional Review Board and Departmental Ethics Committee, in accordance with the ethical principles of the American Psychological Association. Written informed consent to participate in this study was provided by the participants' legal guardian/next of kin. The ninth graders provided their assent, as required by the Chief Scientist in the Israeli Ministry of Education.

## Author Contributions

Both authors conceived, designed, conducted the study, analyzed the data, and wrote the manuscript.

## Conflict of Interest

The authors declare that the research was conducted in the absence of any commercial or financial relationships that could be construed as a potential conflict of interest.

## Publisher's Note

All claims expressed in this article are solely those of the authors and do not necessarily represent those of their affiliated organizations, or those of the publisher, the editors and the reviewers. Any product that may be evaluated in this article, or claim that may be made by its manufacturer, is not guaranteed or endorsed by the publisher.

## Footnotes

^1^The control group began with 27 participants, but three were dropped from the analyses as they did not take part in all parts of the task.

^2^Each full lesson lasted 90 min, of which about 70 min net time was available for team work (the remainder was used for organizing the class, handing out worksheets, etc.).
