# Supplementary material for: Prompting Socially Shared Regulation of Learning and Creativity in Solving STEM Problems
Source: Front Psychol. 2021 Nov 1;12:722535. doi: 10.3389/fpsyg.2021.722535 (PMC8591302; doi:10.3389/fpsyg.2021.722535)
Supplement: Supplementary file 1 [file Image_1.pdf]

Appendix

Sample Problem-Solving Worksheet with Two Types of Support Question Prompts (SSRL and Creativity)

Part A- Forethought

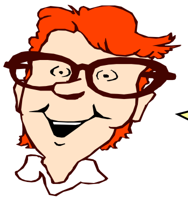

Today you will receive a problem to solve, which may have many possible solutions. Please plan how your team could solve the problem:

How do you plan to work cooperatively in your team?

(Socially Shared Regulation of Learning)

How can you increase the number of ideas to solve the problem?

(Creativity)

Text:

Many villages in developing countries do not enjoy infrastructure for the delivery of fresh water. In some of these villages, the residents need to walk down to a nearby river, fill buckets with water and carry them a few hundred meters back up to a water tank in the village. In many cases, children are involved in this work, which prevents them from attending school. Your team's challenge is to design a product or a system which could help deliver water to the water tank in the village. Your solution cannot depend on electricity or fuel, as there is no regular supply of them to the village.

A.1 What is the problem? Explain it in your own words.

A.2 Identify important features which should be taken into consideration when seeking a solution (e.g., that the product should be safe, cheap, portable etc.).

A.3 Come up with as many solutions as possible. Describe in detail two of your ideas.

A.4 Describe three terms, principles, or phenomena with which you may be familiar from science class that came up in the solutions you suggested.

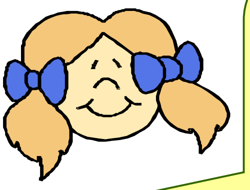

What information is missing? What could help you plan the ideal solution?

(Socially Shared Regulation of Learning)

Were the solutions that your team suggested different from one another?

(Creativity)

Part B- Performance

B.1 Of the ideas you suggested, select the best one. Describe what, in your opinion, makes it the best idea.

B.2 Draw a sketch or write a paragraph to describe your idea. Explain about the different parts of the product or solution, give them names and describe their purpose. What are examples of energy transfers which are taking place in your solution?

Part C- Reflection

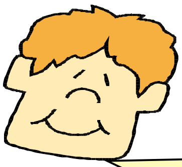

Do you think that there is a better solution than the one you selected? Would you like to replace your solution?

(Socially Shared Regulation of Learning)

What makes your chosen solution an original one?
(Creativity)
